# Metagenomes from High-Temperature Chemotrophic Systems Reveal Geochemical Controls on Microbial Community Structure and Function

**DOI:** 10.1371/journal.pone.0009773

**Published:** 2010-03-19

**Authors:** William P. Inskeep, Douglas B. Rusch, Zackary J. Jay, Markus J. Herrgard, Mark A. Kozubal, Toby H. Richardson, Richard E. Macur, Natsuko Hamamura, Ryan deM. Jennings, Bruce W. Fouke, Anna-Louise Reysenbach, Frank Roberto, Mark Young, Ariel Schwartz, Eric S. Boyd, Jonathan H. Badger, Eric J. Mathur, Alice C. Ortmann, Mary Bateson, Gill Geesey, Marvin Frazier

**Affiliations:** 1 Thermal Biology Institute and Department of Land Resources and Environmental Sciences, Montana State University, Bozeman, Montana, United States of America; 2 J. Craig Venter Institute, Rockville, Maryland, United States of America; 3 Synthetic Genomics Inc., La Jolla, California, United States of America; 4 Center for Marine Environmental Studies, Ehime University, Matsuyama, Japan; 5 University of Illinois, Urbana, Illinois, United States of America; 6 Idaho National Laboratory, Idaho Falls, Idaho, United States of America; 7 Thermal Biology Institute and Department of Plant Sciences and Plant Pathology, Montana State University, Bozeman, Montana, United States of America; 8 Thermal Biology Institute and Department of Microbiology, Montana State University, Bozeman, Montana, United States of America; 9 Department of Marine Science, University of South Alabama, Mobile, Alabama, United States of America; Universidad Miguel Hernandez, Spain

## Abstract

The Yellowstone caldera contains the most numerous and diverse geothermal systems on Earth, yielding an extensive array of unique high-temperature environments that host a variety of deeply-rooted and understudied *Archaea*, *Bacteria* and *Eukarya*. The combination of extreme temperature and chemical conditions encountered in geothermal environments often results in considerably less microbial diversity than other terrestrial habitats and offers a tremendous opportunity for studying the structure and function of indigenous microbial communities and for establishing linkages between putative metabolisms and element cycling. Metagenome sequence (14–15,000 Sanger reads per site) was obtained for five high-temperature (>65°C) chemotrophic microbial communities sampled from geothermal springs (or pools) in Yellowstone National Park (YNP) that exhibit a wide range in geochemistry including pH, dissolved sulfide, dissolved oxygen and ferrous iron. Metagenome data revealed significant differences in the predominant phyla associated with each of these geochemical environments. Novel members of the Sulfolobales are dominant in low pH environments, while other Crenarchaeota including distantly-related Thermoproteales and Desulfurococcales populations dominate in suboxic sulfidic sediments. Several novel archaeal groups are well represented in an acidic (pH 3) Fe-oxyhydroxide mat, where a higher O_2_ influx is accompanied with an increase in archaeal diversity. The presence or absence of genes and pathways important in S oxidation-reduction, H_2_-oxidation, and aerobic respiration (terminal oxidation) provide insight regarding the metabolic strategies of indigenous organisms present in geothermal systems. Multiple-pathway and protein-specific functional analysis of metagenome sequence data corroborated results from phylogenetic analyses and clearly demonstrate major differences in metabolic potential across sites. The distribution of functional genes involved in electron transport is consistent with the hypothesis that geochemical parameters (e.g., pH, sulfide, Fe, O_2_) control microbial community structure and function in YNP geothermal springs.

## Introduction

Metagenome sequencing of microbial community DNA holds tremendous promise for determining the properties of indigenous microbial populations and the composition and structure of microbial communities in natural environments [1]–[6]. Recent studies suggest that metagenome sequencing can be quite effective in characterizing low-complexity sites such as the extremely acidic (pH<1) mine-drainage biofilm at Iron Mountain [7], [8] or deep subsurface (2.8 km) drainage waters from an African gold mine [9]. The extreme geochemical conditions at the Iron Mountain site (i.e., high H^+^, Fe^II^, As^III^) limit the microbial community composition, and metagenomic sequencing has been used to successfully assemble near-complete, consensus genomes of indigenous *Ferroplasma* type II and *Leptospirillum* group II populations. The metagenomic data also provided necessary tools (e.g., expression arrays) for evaluating key genetic determinants important to the function of these organisms in this geochemical context [7], [8]. Specifically, oxidation of Fe^II^, arsenic resistance, and defense against oxidative stress are important genetic attributes of the organisms inhabiting these environments [8].

In more complex microbial communities, significantly greater sequencing is required to obtain adequate depth of coverage for phylogenetic and functional analyses [3], [5]. Surface soils and marine photic zones are among the most diverse environments on Earth [3], [5], [10]–[11], and the gene diversity observed in the *Global Ocean Survey* metagenomes [5] precluded extensive assembly of individual sequence reads into larger contigs and scaffolds. However, the metagenomes clearly revealed dominant organisms important in marine systems, as well as immense diversity and identification of numerous new protein families [5].

The relative simplicity of high-temperature environments as indicated from prior 16S rRNA gene surveys [12]–[15] provides a unique opportunity for utilizing metagenome sequencing to elucidate phylogenetic and functional diversity in model environmental systems. The primary goal of this work was to evaluate the phylogeny and ecology of five disparate chemotrophic microbial communities in Yellowstone National Park (YNP) using environmental shotgun sequencing in the context of extensive geochemical characterization. Our specific objectives were to (i) identify predominant indigenous populations of five high-temperature geothermal microbial communities in YNP using multiple phylogenetic analysis approaches of metagenome sequence data, (ii) determine the metabolic potential of these indigenous microorganisms using bioinformatic and functional analysis of metagenome sequence, and (iii) identify candidate protein-coding genes that may have relevance to variable geochemical conditions across these geothermal systems. The phylogeny of specific functional genes provides direct insight towards the possible role of individual population(s) within each community, and provided candidate genes whose distribution may be a function of major geochemical attributes such as pH, dissolved oxygen, Fe and or S species.

## Results and Discussion

### Geochemical Context of Chemotrophic Geothermal Habitats

**Figure 1:**
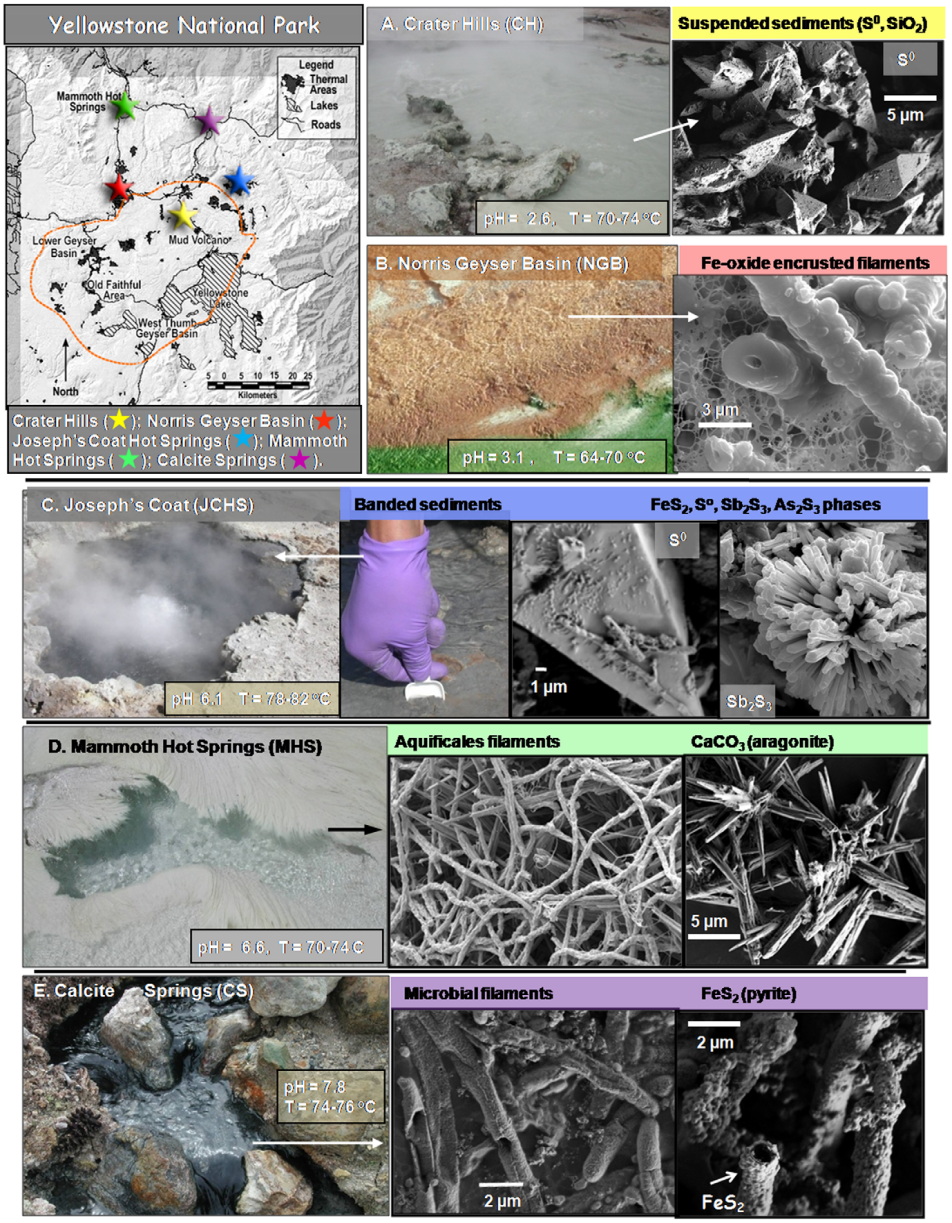
Habitat context and geothermal site characteristics. Site photographs and scanning electron micrographs (SEM) of microbial mats and solid phases associated with each geothermal sample used for metagenome sequencing (map of Yellowstone National Park and site locations shown in top left panel). **A.** Crater Hills (CH, gold); **B.** Norris Geyser Basin (NGB, red); **C.** Joseph's Coat Hot Springs (JCHS, blue); **D.** Mammoth Hot Springs (MHS, green). **E.** Calcite Springs (CS, violet).

The extensive geochemical diversity of terrestrial hot springs in YNP provides a natural laboratory for evaluating the role of specific geochemical variables such as pH, dissolved oxygen, ferrous iron and the presence of sulfide (or elemental S) on the distribution and functional adaptations of thermophilic microorganisms. The five chemotrophic environments chosen for this study encompass a representative range of habitat types characteristic of non-phototrophic high temperature environments in YNP. They exhibit major differences in pH (∼3–8), dissolved oxygen, Fe, total dissolved sulfide, as well as predominant solid phases intimately associated with the microbial community (Figure 1, Table 1). It is hypothesized that geochemical and hydrodynamic attributes of each site control the phylogenetic composition and corresponding functional capabilities of these microbial communities.

**Table 1:** Aqueous geochemical parameters^1^ and predominant solid phases associated with the five geothermal microbial communities sampled for metagenome sequencing.

Location	T	pH	I	DIC	DS	O_2_	As	Fe	CH_4_	H_2_	Solid Phases^2^	Site^3^	Coordinates
	°C		–mM–		------------uM------------				----nM----				
**Crater Hills (CH)**	75	2.5	18	1.3	1–2	<3	2	230	300	67	**S^0^, SiO_2_**	*Alice Spring* CHANN041	44° 39′ 12.108″ N. Lat 110° 28′ 39.6″ W. Lon
**Norris Geyser Basin (NGB)**	65	3.0	17	0.82	<1	50	27	37	300	15	**Fe(AsO_4_)_0.6_(OH)_3_**	*Beowulf Spring* NHSP35	44° 43′ 53.4″ N. Lat 110° 42′ 40.9″ W. Lon
**Joseph's Coat Hot Springs (JCHS)**	80	6.1	23	0.45	25	<3	130	0.7	900	107	**S^0^, Sb_2_S_3_, FeS_2_, As_2_S_3_, SiO_2_**	*Scorodite Spring* JCS083	44° 44′ 21.4 N. Lat 110° 19′ 28.2″ W. Lon
**Mammoth Hot Springs (MHS)**	71	6.6	32	16.5	80	<3	20	0.4	<10	17	**CaCO_3_ (aragonite), S^0^**	*Narrow Gauge* MA041	44° 58′ 9.915″ N. Lat 110° 42′ 35.4″ W. Lon
**Calcite Springs (CS)**	75	7.8	16	0.8	70	<3	18	3.4	<10	30	**FeS_2_, S^0^**	*Scary Spring*	44° 54′ 17.46″ N. Lat 110° 24′ 14.5″ W. Lon

1 I =  ionic strength calculated from aqueous geochemical modeling at sample temperature; DIC  =  dissolved inorganic C; DS = dissolved sulfide; DO =  dissolved oxygen.

2 predominant solid phases determined using scanning electron microscopy (FE-SEM) coupled with energy dispersive analysis of x-rays (EDAX) and x-ray diffraction (XRD).

3 Site  = specific spring name and Yellowstone National Park Thermal Inventory Number (when available, www.rcn.montana.edu).

A brief comparison of the geochemical attributes across these five sites is necessary for evaluating potential functional differences among the numerically dominant phyla identified in each microbial community. The low pH (2.6) turbid pool at Crater Hills (CH) contains suspended particulates (∼1–2 g/L) comprised primarily of elemental S and SiO_2_ (Figure 1a). Dissolved O_2_ values are below detection (∼3 µM), and the low concentrations of other dissolved gases including H_2_S, H_2_ and CH_4_ are characteristic of a steam-dominated, acid-sulfate system [16] (Table 1). In contrast, the acidic (pH 3.1) mat community sampled from a geothermal spring in Norris Geyser Basin (NGB) is from an oxygenated outflow channel (∼65–70°C) dominated by Fe^III^-oxides [17]. The electron and x-ray amorphous Fe-oxides in NGB form as encrustations and nodules around filamentous organisms (Figure 1b) at channel locations where dissolved O_2_ values range from 30–100 µM (20–60% of saturation, Table 1). The Fe-oxide mats at NGB and the S-rich sediments of CH have both been shown to contain significant numbers of crenarchaea within the order Sulfolobales [13], [18]–[19]. Consequently, a comparison between these two sites provides an excellent opportunity to study geochemical factors responsible for functional diversity of different members of these acidophilic crenarchaea.

Although the three higher pH sites at Joseph's Coat (JCHS), Calcite (CS), and Mammoth Hot Springs (MHS) are all sulfidic and sub-oxic, they are geochemically distinct from one another and yield significantly different microbial communities (Figure 1). The anoxic, submerged sediments sampled at JCHS (80°C) are dominated by reduced phases of sulfur including pyrite (FeS_2_), stibnite (Sb_2_S_3_), orpiment (As_2_S_3_) and elemental S (Figure 1c). The aqueous phase of JCHS contains high concentrations of CH_4_, H_2_, NH_4_, arsenite, and thiosulfate (Table 1). Consequently, numerous reduced chemical species could serve as electron donors to support chemolithotrophic metabolism in the absence of O_2_ [13]. The Calcite and Mammoth Springs ‘streamer’ communities were sampled from high-velocity (i.e., ∼0.1–0.3 m s^−1^), highly-sulfidic outflow channels (>80 µM total dissolved sulfide, Table 1), and have been shown to be dominated by microorganisms of the deeply-rooted bacterial Order Aquificales [20]–[22]. Although total soluble Fe is low in the source waters of JCHS and CS, a combination of higher pH (6.1 and 7.8, respectively) and high sulfide results in the deposition of pyrite (FeS_2_) in both environments. At CS, the deposition of pyrite on filamentous cell walls yields characteristic black ‘streamers’ (Figure 1e) that are variably intermixed with thin, but visible coatings of elemental S. In contrast, the predominant mineralization processes at MHS [20]–[21] result in the deposition of CaCO_3_(aragonite) and elemental S to form white, pale yellow ‘streamers’ (Figure 1d). A comparison of the geochemistry and functional gene content across these three sulfidic sites provides a unique opportunity for identifying the role of S in the energetics and metabolism of different thermophilic microorganisms [23]–[24].

### Analysis of Metagenome Sequence

Approximately 14–15,000 sequence reads of an average length of ∼800 bp (∼11–12 Mbp) were obtained for each site (Table S1). The number of individual sequence reads that assembled into contigs and scaffolds (Celera Assembler [5]) varied considerably across these five sites. Though the total amount of sequence obtained per site was relatively small, there was considerable assembly producing scaffolds of significant length (>174 kb). The length of these scaffolds indicates that these communities are dominated by a small number of relatively homogenous microbial species, which facilitated the phylogenetic and functional annotation of these datasets. Coverage estimates for contigs ranged from ∼6.6 at MHS, 5 at CH, and just over 2 at JCHS, CS and NGB (additional assembly statistics in Table S1).

### Archaeal-Dominated Communities (Crater Hills, Norris Geyser Basin, Joseph's Coat)

**Figure 2:**
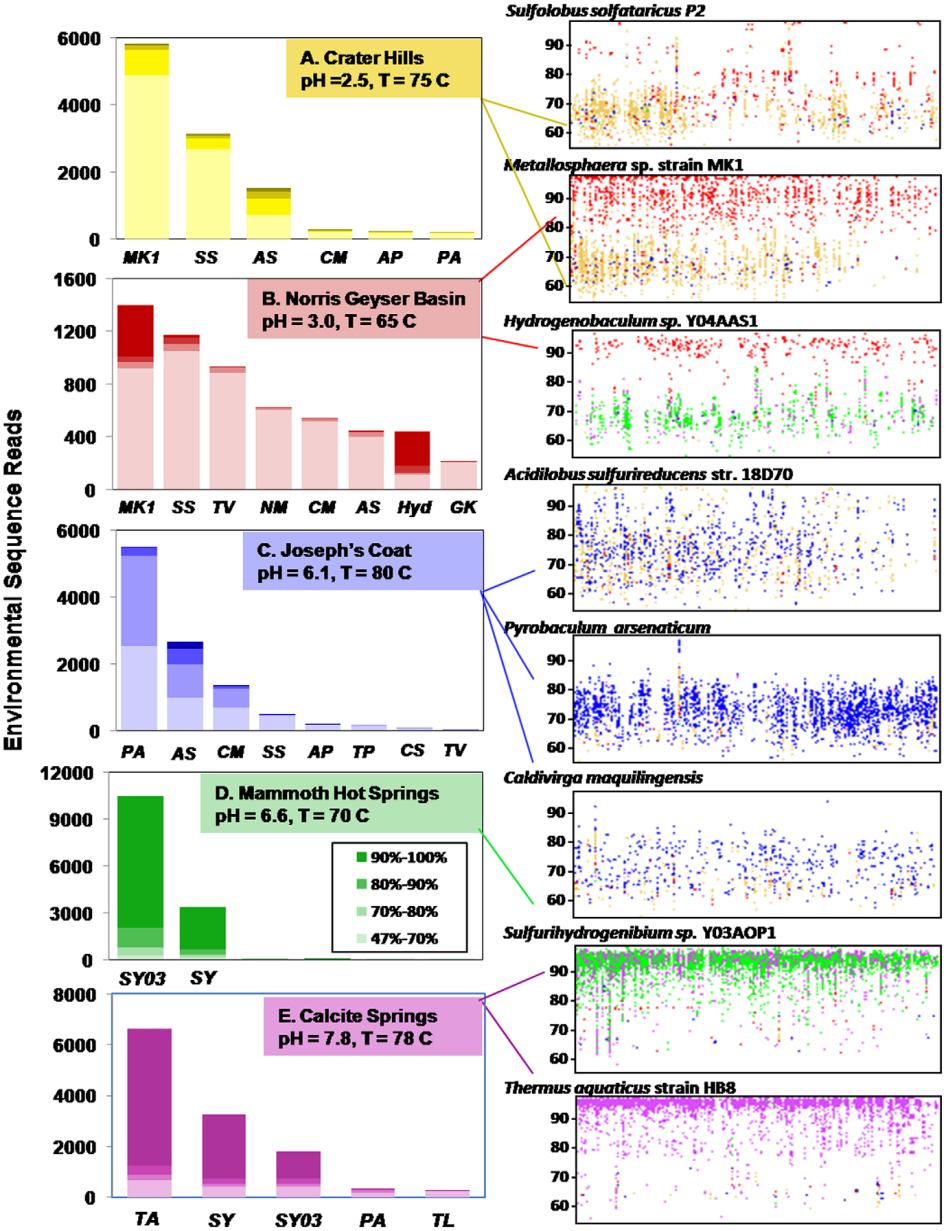
Phylogenetic analysis of metagenome sequence data. Binning of metagenome sequence reads (l**eft column)** from Crater Hills (gold), Norris Geyser Basin (red), Joseph's Coat Springs (blue), Mammoth Hot Springs (green) and Calcite Springs (violet) (with blastn similarity scores (E-values) of <10^−10^) to closest reference microbial genomes (abbreviations below). Environmental sequence reads were further categorized based on nucleotide identity ranging from 47–100% (shaded from light to dark, legend shown only for MHS). Fragment recruitment (r**ight column)** of metagenome sequence reads to reference microbial genomes is plotted across each reference genome (x-axis) at a nucleotide identity ranging from 50–100% (y-axis). Reference genomes: **MK1** = *Metallosphaera* sp. str. MK1 (partial genome sequence) [19]; **AS** *= Acidilobus sulfurireducens* (partial genome sequence) [25]; **SS** *= Sulfolobus solfataricus* [26]; **CM** *= Caldivirga maquilingensis* [34]; **AP** *= Aeropyrum pernix* [27]; **PA** *= Pyrobaculum arsenaticum* [38]; **TV** *= Thermoplasma volcanium* [31]; **NM** *= Nitrosopumilus maritimus* [32]–[33]; **Hyd** *= Hydrogenobaculum* sp. Y04AAS1 [35]; **GK** *= Geobacillus kaustophilus* [36]; **TP** *= Thermofilum pendens*; **CS** *= Caldicellulosiruptor saccharolyticus* [37]; **SY03** *= Sulfurihydrogenibium* sp. Y03AOP1 [35], [39]; **SY** *= Sulfurihydrogenibium yellowstonensis* [35], [39]; **TA** *= Thermus aquaticus* Y5.1 MC23; **TL** *= Thermotoga lettingae* [40].

Analysis of individual sequence fragments (e.g. ∼800 bp) from Crater Hills (CH) using binning and fragment recruitment approaches both reveal that the majority of sequence reads (∼60%) are phylogenetically related to crenarchaea within the order Sulfolobales, and that a smaller number of sequence reads are more closely related to members of the Desulfurococcales (∼12%) and Thermoproteales (∼5%). The partial genome sequence of *Acidilobus sulfurireducens* str. 18D70, an anaerobic, S-respiring Desulfurococcales isolated from YNP [25] recruits approximately 10% of the sequence reads from CH (Figure 2a). However, none of the current genome sequences (e.g., *Sulfolobus solfataricus* [26], *Aeropyrum pernix* [27], *Hyperthermus butylicus* [28] and *Staphylothermus marinus* [29]) are good references for organisms present at this site, as evinced by nucleotide identities generally less than 70%, and inconsistent coverage relative to reference genomes (Figure 2a). 16S rRNA gene sequences observed in the metagenome data are consistent with prior 16S rRNA gene analysis at CH using PCR and clone library analysis (Figure S1). One near full-length 16S rRNA gene observed in the assembled sequence data (Table S2) was present in a large scaffold of 165,334 bp, and this novel taxa (91% nucleotide identity to *S. solfataricus*) represents an important Sulfolobales population in CH.

The acidic Fe^III^-oxide microbial mat from Norris Geyser Basin (NGB) is the most diverse archaeal community of the five sites included in this study, and contains several novel lineages within the current Phylum Crenarchaeota, Candidate Phylum Thaumarchaeota [30] and Phylum Euryarchaeota (Figure 2b). A significant fraction (∼11%) of the metagenome sequence reads exhibit reasonable nucleotide identity (80–99%) to the available genomic sequence (∼2200 ORFs) of *Metallosphaera* sp. str. MK1 [19], representing a coverage of ∼0.3x (Figure 2b). Additional Sulfolobales-like sequence reads (∼9%) do not recruit well to current reference genomes, and exhibit low nucleotide identity to *S. solfataricus* P2 [26] (Figure 2b). Approximately 8% of the total sequence reads are related to a novel euryarchaeal lineage, and ∼5% are related to organisms within the candidate Phylum Thaumarchaeota [30]. These sequences are contributed by indigenous organisms distantly related to currently cultivated relatives, and exhibit low nucleotide identity (47–70%) to reference genomes within the Thermoplasmatales (i.e., *Thermoplasma volcanium*, [31]) and Nitrosopumilales (i.e., *Nitrosopumilus maritimus, Nitrosocaldus yellowstoni* [32]–[33], respectively). Novel members of the Thermoproteales (∼5%) and Desulfurococcales (∼5%) are also present in the Fe-oxide mats. Currently, the best references for these sequence reads include *Caldivirga maquilingensis* [34] and the partial genome sequence data of *Acidilobus sulfurireducens* [25], respectively (Figure 2b). A smaller subset of environmental sequence reads (∼3%) from NGB show excellent identity to the genome of *Hydrogenobaculum* sp. Y04AAS1 [35] (Figure 2b), and although the estimated coverage of this genome is only 0.2x, the number of high-identity (∼90%) sequence matches, as well as the thorough distribution of fragments across the reference genome, suggests that highly similar *Hydrogenobaculum*-like organisms are important members of the Fe-mat community. Other minor (<0.5% of sequence reads) bacterial populations present include distant relatives of *Geobacillus kaustophilus* [36] and *Caldicellulosiruptor saccharolyticus* [37].

Metagenome sequence from Joseph's Coat Hot Springs (JCHS) is largely contributed by indigenous members of the Thermoproteales and Desulfurococcales (Figure 2c). The largest fraction of sequence reads (∼40%) ‘bin’ nearly equivalently to the *Pyrobaculum* spp. genomes [38] (i.e., *P. aerophilum*, *P. arsenaticum* DSM 13514, *P. caldifontis* JCM 11548). Recruitment of sequence reads to these reference organisms shows similar nucleotide identities (60–80%) across the genomes of *Pyrobaculum* spp. and *Thermoproteus neutrophilus*, (Figure 2c) representing significant and fairly uniform coverage (∼1x). A second Thermoproteales population in JCHS (∼10% of sequence reads) is more closely related to *Caldivirga maquilingensis* [34] (Figure 2c), while approximately 20% of the metagenome sequence reads show homology to the partial genome sequence (∼2200 ORFs) from *A. sulfurireducens* str. 18D70 [25]. As found in Crater Hills, these sequences do not show significant nucleotide identity to currently available Desulfurococcales genomes such as *A. pernix* (not shown). Phylogenetic assignment of 16S rRNA genes observed within the metagenome sequence data from JCHS (Table S2) is consistent with prior identification of 16S rRNA genes using PCR and cloning (Figure S1).

### Bacterial-Dominated Communities (Mammoth Hot Springs and Calcite Springs)

A large majority (∼90%) of metagenome sequence reads from the streamer community at Mammoth Hot Springs (MHS) were highly similar (nucleotide identity >90%) to the genome of *Sulfurihydrogenibium* sp. Y03AOP1 [35], isolated from Obsidian Pool, YNP (Figure 2d). The fact that this microbial community is dominated by a single bacterial population(s) within the Aquificales suggests that this population is highly adapted to the geochemical and hydrological attributes of this environment. Significant and relatively uniform coverage (∼3.9x) of either of the two reference *Sulfurihydrogenibium* genomes (strain Y03AOP1 or *S. yellowstonensis*, [35]) was obtained from this site with only 12 Mbp of random sequence.

Metagenomic sequence from the higher pH (7.8), sulfidic site at Calcite Springs (CS) is also largely bacterial, exhibiting high nucleotide identity to genome sequences of *Thermus aquaticus* and *Sulfurihydrogenibium yellowstonensis* SS-5, which was isolated from this site [39]. These two bacterial populations represent a major fraction of the metagenome sequence from this community (∼45% *Thermus* and ∼36% *Sulfurihydrogenibium*), resulting in ∼1.5x and 1.7x coverage relative to the reference genomes of *T. aquaticus* and *S. yellowstonense* (or strain Y03AOP1), respectively (Figure 2e). A small fraction (<1–2%) of sequence reads from CS are similar to *Pyrobaculum*-like organisms (the dominant archaea in this system) as well as bacterial population(s) distantly related to genome sequence of *Thermotoga lettingae* [40] (Figure 2e).

### Predominant Sequence Assemblies

**Figure 3:**
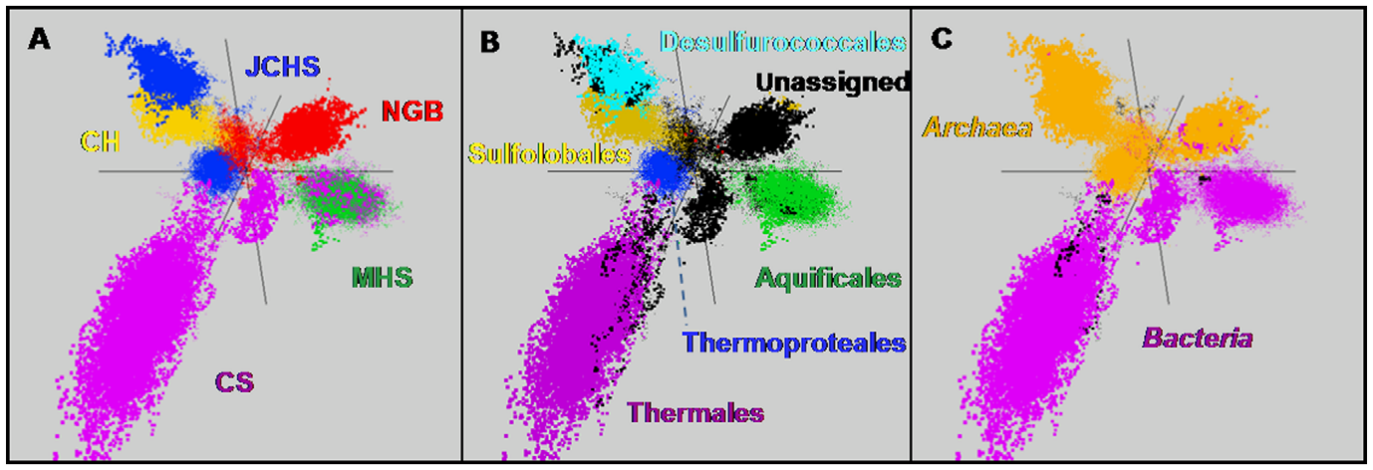
Nucleotide word frequency plots and phylogenetic analysis of metagenome assemblies. Nucleotide word frequency principal component analysis (PCA) of assembled metagenome sequence data (contigs>1500 bp) from five chemotrophic geothermal habitats in YNP: **A.** Metagenome sequence colored by site (Crater Hills  =  gold, Norris Geyser Basin  =  red, Joseph's Coat  =  blue, Mammoth Hot Springs  =  green, Calcite Springs  =  violet). **B.** Identical PCA orientation of metagenome sequence observed in *Panel A*, but colors now designate phylogenetic affiliation at the order level (Sulfolobales  =  gold; Desulfurococcales  =  light blue; Thermoproteales  =  dark blue; Aquificales  =  green; Thermales  =  violet; Unassigned  =  black), and **C.** Identical PCA orientation with phylogenetic classification at the domain-level (*Archaea  = * gold, *Bacteria*  =  violet).

Principal components analysis (PCA) of nucleotide word frequencies (1–5) derived from the assembled libraries from each site was used to cluster and classify the metagenome assemblies (Figure 3). When contigs >1500 bp from all sites are included together in this analysis, the individual sites separated into distinct and highly-constrained clusters (Figure 3a). The distinct sequence signatures across sites reflect differences in the dominant phyla present within these five different geothermal habitats. The PCA plot was then fixed in this orientation, and sequences classified phylogenetically (Figure 3b). Distinct, but partially overlapping Sulfolobales sequence assemblies (gold) are evident in the acidic, elemental sulfur site (CH) and the acidic Fe-oxide mats (NGB). The anaerobic, sulfidic sediments of Joseph's Coat Springs (JCHS) are dominated by two major sequence assemblies that are most similar to reference genomes within the crenarchaeal orders Thermoproteales and Desulfurococcales (blue, Figure 3b). Bacterial sequences within the order Aquificales dominate the carbonate ‘streamer’ communities (70–72°C) at MHS, and are also one of the dominant phyla present in the pyritic ‘streamers’ from CS. These scatter plots are presented in three dimensions thereby providing better separation of different clusters, which can only be fully appreciated by interacting with the data directly (nucleotide word frequency plots can be accessed at http://gos.jcvi.org/openAccess/scatterPlotViewer.html).

### Viral Sequences

Metagenome data from all five sites was analyzed for sequences exhibiting significant similarity to known viruses. A small subset of sequence reads (∼1%) from CH, JCHS, and NGB recruit to crenarchaeal virus genomes at nucleotide identities ranging from 50–90%. The majority of virus-like sequences at JCHS (∼70%) were related to *Pyrobaculum Spherical Virus* (PSV), and of 150 viral-like sequence reads, 112 were assembled into larger contigs and scaffolds resulting in an average coverage of 2.5x across the reference PSV genome (Figure S2). The metagenome sequence data suggest that PSV was present in the sediment used to extract DNA, or present in host cells. Recent work also suggests possible assembly of *Pyrobaculum*-like viruses from thermal springs in YNP [41]. The majority of virus-like sequence reads from CH and NGB were most closely related to the reference genomes of Sulfolobales viruses including *Acidianus* two-tailed virus [42], *Stygiolobus* rod-shaped virus [43] and other miscellaneous *Sulfolobus* viruses [18], [44]. However, the viral-related sequence reads from CH and NGB do not assemble into larger contigs or scaffolds and do not result in uniformly random coverage across the viral genomes (data not shown).

### Functional Analysis of Metagenome Sequence

One of the premises of this study is that the metabolic attributes of microbial populations in high-temperature, geothermal environments are influenced by geochemical parameters [45], [46], a subset of which can also be influenced by physical processes such as velocity, turbulence and gas exchange. Following, the genes required for specific physiologies of the populations inhabiting these springs should reflect the geochemical constraints defining these microenvironments (e.g., the presence of specific electron donors and or acceptors). To examine this tenet in an unbiased manner, we first performed a thorough and integrated statistical analysis of metagenome sequence data to identify the biochemical pathways and functions that are utilized differentially in the five microbial communities.

### Metabolic Pathway Reconstruction

**Figure 4:**
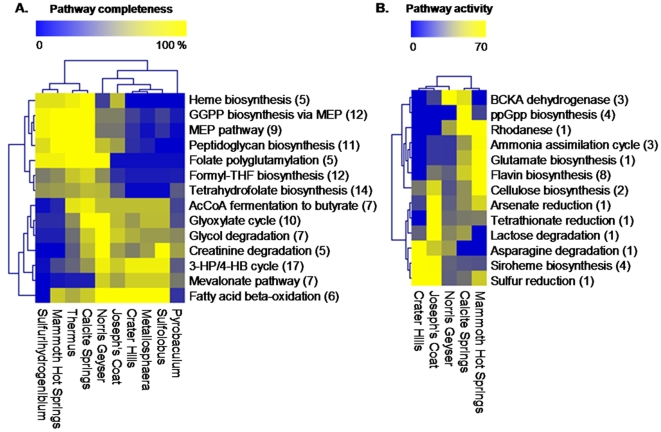
Functional gene analysis. Two-way clustering of biochemical pathways that contributed most to the variability between sites based on PCA analysis. **A.** Comparison of metagenomes and relevant reference genomes based on pathway completeness. Reference genomes: *Sulfolobus solfataricus* P2, *Metallosphaera sedula* DSM 5348, *Pyrobaculum arsenaticum*, *Thermus aquaticus* Y5.1 MC23, *Sulfurihydrogenibium* sp. YO3AOP1. **B.** Comparison of metagenomes based on the median number of blast hits to enzymes in a pathway on a log scale. Abbreviations: MEP, methylerythritol phosphate; GGPP, geranylgeranyl-diphosphate; THF, tetrahydrofolate; 3-HP/4-HB, 3-hydroxypropionate/4-hydroxybutyrate; BCKA, branched-chain keto-acid.

We explored differences and similarities in metabolic pathways among the five communities using a custom metabolic pathway reconstruction database created based on metagenome sequence from each site as well as reference genomes and databases [47]–[49](see Methods). Pathway completeness scores for all MetaCyc [47] pathways found in at least one community (or reference genome) were first subjected to principal component analysis (PCA) to identify pathways that contribute most to variability across the genomes/metagenomes (Figure S3). The completeness data for the key pathways identified by PCA analysis was then clustered using average linkage hierarchical clustering (Figure 4a). As expected, the archaeal and bacterial sites are readily separated at the functional level, and the reference genomes group with the relevant metagenomes (Figure 4a). Results from functional analysis converge with phylogenetic analysis (Figure 2–3) and reveal the importance of distinct phyla in each site (e.g. Sulfolobales in CH; Aquificales in MHS). Although contributions from specific phyla are clearly recognizable, the more diverse communities (especially the acidic Fe-oxide mats) are functionally relatively distant from the major reference species. The major pathways that contribute to differentiating the sites follow the division between bacteria and archaea-specific pathways (Figure 4a). For example, bacteria synthesize terpenoid compounds through the MEP pathway whereas archaea use the mevalonate pathway. The pathway responsible for autotrophic metabolism recently characterized in *Metallosphaera sedula* [50] was clearly represented in the sites dominated by archaea (CH, JCHS, NGB). While the pathway completeness-based analysis can highlight major functional differences between metagenomes and reference genomes, it does not account for the abundance of specific genes, and thus does poorly in differentiating relatively similar communities (e.g. the three archaeal communities).

To assess the similarity of the five communities in terms of abundance of specific genes (i.e., pathway activity), we performed the same analysis outlined above using the median number of blast hits to proteins in each pathway for each site. Results from this analysis, which accounts for differences in organism abundance within sites provide examples of the role of specific electron donors or acceptors (e.g., sulfur, tetrathionate, arsenate) across the three archaeal environments (Figure 4b). Functional analysis provides clues to the types of metabolic potential represented in metagenomes and genomes, but differentiating putative function within protein families based on analysis of partial protein sequences is challenging. For this reason, we also analyzed the genes related to the use of electron donors and acceptors (including those identified in Figure 4a) and CO_2_ fixation in greater detail using assembled metagenome sequence.

### Carbon Fixation and Energy Cycling: Linkage to Geochemistry

To assess differences in the potential metabolism of organisms found within sites exhibiting disparate geochemistry, we searched the five metagenomic assemblies using query sequences (Table S3) of proteins associated with specific autotrophic (CO_2_ fixation) pathways and electron transfer processes involving C, Fe, S, As, N, H_2_ or O_2_ (Table 2). In contrast to the analysis using generic EC-number/reaction associated sequences from public databases described above, the query sequences used for detailed functional analysis were selected from reference organisms that are phylogenetically related to members of these environments (Table S3).

**Table 2:** Identification of metagenome sequences associated with CO_2_ fixation, electron transfer reactions and detoxification across five high-temperature chemotrophic systems in Yellowstone National Park (YNP).

Process	Substrate	Marker Gene^1^	Number of Probable Sequence Matches^2^ in Site				
			*Crater Hills*	*Norris Geyser Basin*	*Joseph's Coat HS*	*Mammoth Hot Springs*	*Calcite Springs*
CO_2_ fixation (reductive TCA)	CO_2_	*aclB*	0	0	0	**1**	**1**
CO_2_ fixation (reductive acetyl-coA)	CO_2_	*acs, cooS*	0, 0	0, 0	0, 0	0, 0	**2, 1**
CO_2_ fixation (3-HP/4-HB) 3	CO_2_	*4hcd, mcm*	**2, 3**	**3, 8**	**4, 2**	0, 0	0, 0
Thiosulfate oxidation	S_2_O_3_^2−^	*tqoAB*	**2**	**2**	**1**	0	0
Oxidation of reduced S	S^2−^	*sqr*	**4**	**10**	**6**	**2**	**3**
Sulfite oxidation	SO_3_^2−^	*sox*	**1**	**3**	**3**	**1**	**1**
Sulfite oxidation	SO_3_^2−^	*soxC*	0	0	0	0	**4**
Hydrogen oxidation (Group 1 Ni-Fe Hyd)	H_2_	*hynS, hynL-like*	**3, 1**	0, 0	**1, 3**	0, 0	0, 1
Arsenite oxidation	As^III^	*aroA*	0	**1**	**1**	0	0
Terminal oxidation^4^	O_2_	*doxB*	**3**	**5**	**1**	0	0
Terminal oxidation	O_2_	*aoxB*	0	**10**	0	0	0
Terminal oxidation	O_2_	*foxA*	0	**4**	0	0	0
Terminal oxidation	O_2_	*other*	0	**1**	0	**2**	**2**
Dissimilatory sulfur reduction^5^	S^0^, S_n_^x−^	*sreA-like* 3	**2**	0	**10**	**2**	**7**
Dissimilatory sulfate reduction	SO_3_^2−^	*dsrA*	0	0	**3**	0	**1**
Dissimilatory N oxide reduction	NO/N_2_O	*norB, nosZ*	0, 0	0, 0	**2, 0**	0, 0	0, 0
Dissimilatory nitrate reduction	NO_3_^−^	*narG*	0	**2**	**4**	0	**2**
Arsenic detoxification	As^III^, As^V^	*arsB, arsC*	**3, 0**	**8, 0**	**5, 0**	**1, 1**	**4, 4**
Mercury detoxification	Hg	*merA*	**2**	**3**	**1**	0	0

1 marker genes code for proteins with high specificity for possible pathway.

2 number of different ‘high-confidence’ sequence matches to marker genes (see Supplementary Table S4 for details on individual sequence matches.

3 3-HP/4-HB  = 3-hydroxypropionate/4-hydroxybutyrate pathway; terminal oxidation = reduction of O_2_ via heme Cu oxidase.

4 includes Mo-pterin proteins similar to *sre*A and *arr*A.

5 no gene sequences with homology to *sox*B, *sox*M, *nir*K, *nir*S, *nap*A, *mcrA*, or *amo*A genes were noted (gene symbols also described in Table S3).

An inventory of genes known to be involved in the five major chemotrophic CO_2_ fixation pathways reveals major differences across sites (Table 2), consistent with the dominant phyla found in each habitat. Genes coding for the key enzyme required for CO_2_ fixation via the reverse tricarboxylic acid (rTCA) cycle (ATP citrate lyase, *acl*B [51]) were observed only in MHS and CS; both metagenome sequences show excellent identity to the *acl*B gene annotated in *Sulfurihydrogenibium* spp. genomes (NCBI). The high-pH (7.8), pyritic mat at Calcite Springs (CS) is the only community to contain evidence of the reductive acetyl-CoA pathway for CO_2_ fixation (marked by genes *acs* of the acetyl-CoA decarboxylase/synthase complex and *coos*, the carbon monoxide dehydrogenase catalytic subunit [52]). These sequences are phylogenetically related to members of sub-dominant populations of Delta-proteobacteria and Firmicutes. Genes specific to the recently reported 3-hydroxyproprionate/4-hydroxybutyrate CO_2_ fixation pathway (4-hydroxybutyryl-CoA-dehydratase, methylmalonyl-CoA-mutase) in *Metallosphaera sedula* [50] were found in sites dominated by archaea (CH, JCHS and NGB). The majority of environmental sequence hits to genes in this pathway were related to Sulfolobales reference genomes, consistent with the dominant phyla observed in CH and NGB, as well as a minor Sulfolobales population in JCHS (Figure 2).

### Possible Chemotrophic Metabolism: Evaluation of Electron Donors and Acceptors

Genes responsible for a sulfide-quinone reductase (SQR, glutathione reductase family of flavoproteins [53]–[54]) were identified in all sites (Table 2), and this is consistent with the presence of dissolved sulfide and other forms of reduced S (e.g. elemental S) in these geothermal environments (Table 1, Figure 1). The environmental *sqr* sequences exhibit closest matches to expected phyla for each site including members of the Sulfolobales (sites CH, NGB, JCHS), the Thermoproteales (site JCHS), the Aquificales (sites CS, MHS) and the Thermales (site CS). An additional sulfur oxidation pathway (*sox* gene cluster [55]–[56]; not to be confused with *sox*-type terminal oxidases, to be discussed below) was observed in CS (one *Thermus*-like and one *Sulfurihydrogenibium*-like sequence) as inferred by the presence of the *sox*C gene. Genes coding for the oxidation of thiosulfate (via the membrane bound *tqo*AB subunits [55]–[57] were noted in sites containing Sulfolobales (CH, NGB, and to a lesser extent JCHS), but not in sites dominated by Aquificales (MHS, CS). Genes coding for Group 1 membrane-bound Ni-Fe hydrogenases [58] were observed primarily in anoxic sulfidic sites containing Sulfolobales and Thermoproteales (CH and JCHS) (Table 2), with several sequences showing significant amino acid identity to the *Acidianus ambivalens* Ni-Fe hydrogenase thought to be linked with a membrane-bound, sulfur-reductase (SreA) [59]–[60]. Arsenite oxidase genes (*aro*A) were observed in two of the five sites (NGB and JCHS), and arsenite oxidation has been measured in both of these systems [61]–[62]. However, it is yet unclear whether arsenite-oxidizing organisms (including the Aquificales) derive energy from catalyzing this exergonic reaction [63]. No evidence of genes responsible for the synthesis of key enzymes in ammonium oxidation (*amoA*) or methanotrophy/methanogenesis (*mcr*A) [64]–[65] were found in the assembled metagenome sequence (not shown in Table 2), suggesting that these may not be dominant microbial processes in the habitats studied here.

**Figure 5:**
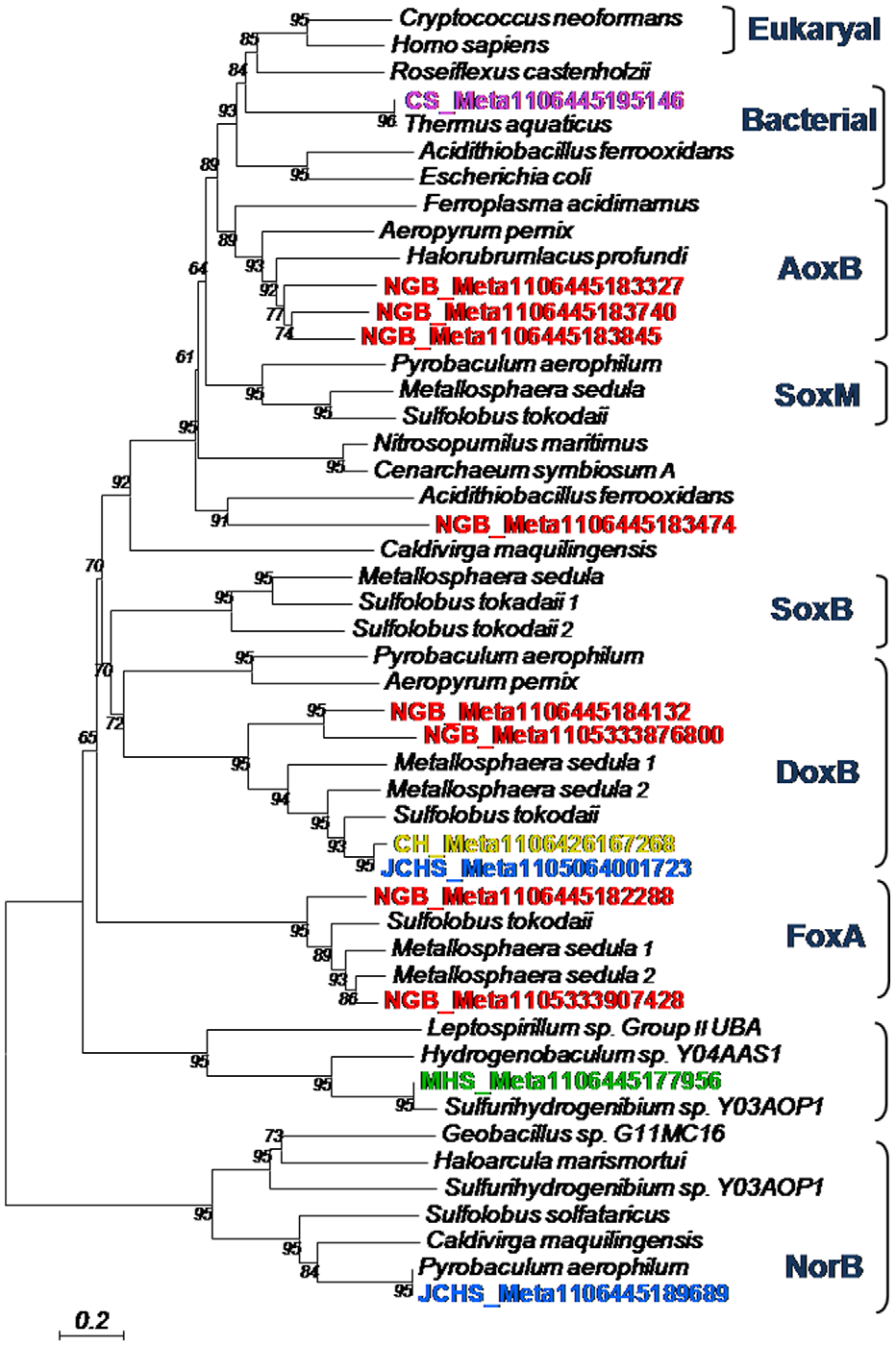
Diversity of heme copper oxidases present in metagenome sequence data. Phylogenetic tree (deduced protein sequences) of heme Cu oxidases and their relationship to nitric oxide (NO) reductases (NorB). Metagenome sequences observed across the five sites are included *(Site_Meta*). All other entries are from annotated genomes found on NCBI. [notations for heme Cu oxidases: AoxB  = *A. pernix* [27]; SoxB, SoxM  =  *S. acidocaldarius* [68], [70]; DoxB  =  *A. ambivalens* [69]; FoxA  =  *M. sedula* [71], [73] and NorB  =  nitric oxide reductases. Tree  =  distance tree created with MEGA using the neighbor-joining method with 100 bootstraps].

Heme Cu oxidases (subunit 1 of terminal oxidase complexes) catalyze the reduction of O_2_ to H_2_O and are an excellent indicator of the potential for aerobic metabolism (or in certain cases, O_2_ detoxification) [66]–[67]. Genes coding for a subunit 1 were identified in all sites, but the diversity and number of gene copies was especially extensive in the Fe-oxide mat of NGB (Figure 5, Table 2). Over 20 different gene sequences matching different types of terminal oxidases were identified in the Fe-oxide mat including *aox*B, *sox*M, *dox*B and *foxA*- like cytochrome c oxidases [27], [68]–[71]. The abundance and diversity of terminal oxidase genes is consistent with the observed O_2_ influx in this outflow channel habitat [17], [61], [72]. Four different copies of the *fox*A gene observed in the Fe-mats are all related to *Metallosphaera* spp. sequences, and is consistent with observations suggesting that this terminal oxidase is utilized when Fe^2+^ serves as an electron donor [71], [73]–[74]. The complete absence of this gene in the other four sites is also consistent with the fact that the oxidation of Fe(II)is not a dominant process in the sulfidic habitats.

The only heme Cu oxidase genes found in CH and JCHS were *dox*B-like sequences with ∼70% amino acid identity to other Sulfolobales and Thermoproteales reference genomes, respectively (Figure 5, Table S4). Recent evidence suggests that the terminal oxidase complex containing *doxB* is utilized when reduced S species (e.g., elemental S) serve as the electron donor [57], which is consistent with the fact that both CH and JCHS contain dissolved sulfide, elemental S, and are sub-oxic (Figure 1, Table 1). The sites dominated by bacteria (MHS and CS) each contain two sequences matching the sub-unit I of cytochrome c oxidases annotated in the *Sulfurihydrogenibium* and *Thermus* spp. reference genomes, respectively (Figure 5, Table S4).

Other terminal electron acceptors besides O_2_ that may be important in these microbial habitats include nitrate, ferric iron, arsenate, thiosulfate, elemental S, sulfate or CO_2_. Gene sequences similar to putative molybdenum (Mo)-pterin subunit I arsenate reductases (*arr*A) [75]–[76] were abundant in JCHS (Figure 4b, Table 2), and this correlates with the high As concentrations at this site. However, genes coding for dissimilatory nitrite reductases (*nir*K, *nir*S) [77], ferric iron reductases (*fer*) [78]–[79] and methyl coenzyme M reductase (*mcr*A) [65] were not observed in the metagenome data. Interestingly, genes coding for known dissimilatory nitrate reductases (*nar*G) and nitric oxide reductases (*nor*B) were found in JCHS (Table 2, Figure 5) and show excellent identity (E-values<10^−63^) to those annotated in the *Pyrobaculum* spp. genomes [79]. Based on current models of dissimilatory nitrate reduction in bacteria [77], [80]–[81], a nitrite reductase (*nir*K or *nir*S) would be required to produce NO, which serves as a substrate for nitric oxide reductase (*nor*B) to produce N_2_O. Nevertheless, based on currently known gene function, the indigenous *Pyrobaculum*-like populations exhibit partial metabolic potential for denitrification. The nitrate reductase genes (*nar*G) found in NGB were affiliated with sub-dominant phyla within the bacterial order Bacillales, while those in CS were affiliated with *Thermus*-like organisms.

Genes coding for putative sulfur reductases (SreA-like) were observed in all habitats that contain reduced forms of sulfur (CH, JCHS, MHS, CS; Table 2, Figure 4b). The exact function of these putative Mo-pterin proteins remains to be elucidated, but work with related proteins in *Acidianus ambivalens*, *Aquifex aeolicus* and *Pyrococcus furiosus* (representing several thermophilic groups) suggests that a membrane-bound SreA (Mo-S binding site) acts to transfer electrons to elemental S [60], [82]. In several cases, H_2_ can serve as the electron donor as has been noted in *A. ambivalens* and *P. furiosus* [60], [83]–[84].

Genes known to be involved in dissimilatory sulfate reduction including *dsr*A (codes for the sulfite reductase subunit [85]) were only observed in JCHS and CS. The phylogenetic identity of *dsr*A genes (3) observed at JCHS suggests that the indigenous relatives of *Pyrobaculum* spp. and or *Caldivirga* spp. exhibit metabolic potential for sulfate reduction, in addition to the possible reduction of more reduced forms of S (i.e., *sre*A-like discussed above). The *dsr*A sequence from CS is contributed from a relative of the less-dominant deltaproteobacterial population(s) present at this site [e.g. *Desulfovibrio* and or *Desulfococcus*-related sequence matches; Table S4).

### Trace Element Detoxification

All sites contained evidence of *ars*B genes (Table 2), which code for efflux proteins used to transport arsenite out of the cell under toxic conditions [86]–[87]. The high arsenic concentrations associated with Yellowstone's geothermal ecosystem may necessitate that these microorganisms be capable of efficient arsenite efflux (aqueous As levels ranged from 10 to 130 µM across the sites discussed here; Table 1). The *ars*C gene, which is often found together with *ars*B on the *ars* operon [87] and codes for an arsenate reductase associated with detoxification, was only found in the bacterial dominated sites (MHS, CS), and these sequences were affiliated with *Thermus* and *Sulfurihydrogenibium-*like organisms. Genes that code for the mercuric reductase (*mer*A) used in Hg detoxification [88]–[90] were found in sites dominated by archaea (CH, NGB, JCHS); all *mer*A sequences (5 total) were most closely related to genome sequences from the Sulfolobales (Table S4). Mercury and arsenic are two of the most important toxic constituents originating from Yellowstone's geothermal features [91]–[93], and it is noteworthy that the deeply-rooted phyla present in these environments exhibit potential for the detoxification of these elements.

### Summary

Phylogenetic and functional analysis of random shotgun sequence data from five different geothermal environments ranging in pH from 2.5 to 7.8 suggest that these microbial communities are composed of numerically predominant microbial populations whose functional attributes are consistent with geochemical conditions. The two acidic sites (CH, NGB) and the near-neutral sulfidic site (JCHS) were dominated by sequences belonging to members of the *Archaea*. In contrast, the two microbial ‘streamer’ communities were dominated by sequences belonging to the *Bacteria*, including organisms within the deeply-rooted bacterial lineages of Aquificales (MHS, CS) and Thermales (CS). High-temperature springs with pH less than ∼6 were dominated by archaea (although *Hydrogenobaculum*-like organisms are important in NGB), whereas sites with pH values above ∼6 were dominated by bacteria. In addition, the distribution of different archaeal sequence reads from pH 2.5 to 7.8 confirmed the importance of Sulfolobales relatives at low pH (2.5 and 3.0 at CHAS and NGB), compared to Thermoproteales relatives at near-neutral pH and above (6.1 at JCHS). However, all three archaeal-dominated sites contained a significant number of contigs corresponding to novel Desulfurococcales populations. Moreover, a modest number of Sulfolobales sequences were observed in JCHS at a pH of 6.1. Consequently, the metagenome data show that members within the Class Thermoprotei commonly co-occur in the archaeal communities of YNP, and that the relative abundance of specific members of this Class change across sites with major differences in pH and or the presence of dissolved oxygen (Table 1).

The sequencing depth of indigenous populations (estimated based on coverage of reference genomes) varied significantly across the five geothermal sites, tracking inversely with microbial diversity. For example, environmental sequence data from the lowest diversity site (MHS) provided ∼4x coverage relative to the *Sulfurihydrogenibium* sp. Y03AOP1 or *S. yellowstonensis* genomes. At the other extreme, the 65°C, Fe^III^-oxide mat (NGB) exhibited considerable diversity including several novel archaeal populations within the Crenarchaeota (e.g. Sulfolobales, Desulfurococcales, Cenarchaeales, other uncharacterized Groups within the Crenarchaeota) and the Euryarchaeota, as well as a dominant bacterial population(s) of *Hydrogenobaculum*-like organisms, acidophilic members of the Aquificales [22]. Consequently, the sequencing depth of the dominant organisms in NGB (<1x coverage for *Metallosphaera* sp. str. MK1 and *Hydrogenobaculum* sp. Y04AAS1) is considerably lower than the other sites. Analysis of this sequence diversity is also compounded by the fact that the archaea present in NGB (as well as CH and JCHS) are only distantly related to organisms whose genomes have been sequenced to date, and in some cases represent order-level (or higher) lineages that do not yet have a cultured representative.

With the rapid decline in sequencing costs, and the adoption of new pyrosequencing technologies, the amount of sequence coverage reported here is modest. However, our results indicate that modest metagenome sequencing in high-temperature geothermal environments provides an excellent tool for assessing and characterizing the predominant members of these microbial communities, as well as the possible functional attributes of these indigenous populations. This study was initiated as the first phase of a more extensive project (DOE-Joint Genome Institute Community Sequencing Project) aimed at characterizing the prokaryotic gene diversity found within phototrophic and chemotrophic geothermal sites of YNP. Consequently, further sequencing results will focus on building consensus genomic content of predominant indigenous and novel microorganisms in geothermal chemotrophic environments, and expand the inventory and analysis of functional attributes important for the survival and growth of these extremophiles.

## Materials and Methods

### Sampling Sites

Five geothermal microbial communities in Yellowstone National Park (Figure 1) were sampled during summer-fall 2006 at the following research sites that have been subject to significant prior characterization: Crater Hills-*Alice Spring* (CH), Norris Geyser Basin- *Beowulf Spring* (NGB); Joseph's Coat Hot Springs-*Scorodite Spring* (JCHS); Mammoth Hot Springs-*Narrow Gauge* (MHS); Calcite Springs-*Scary Spring* (CS) [13], [17], [18], [20], [21], [22]. The sites were chosen to represent a breadth of geochemical conditions and thermophilic phyla, and to focus exclusively on chemotrophic communities ranging in pH from 2.5 to 7.8 (Table 1). The location, physical and geochemical features of each sampling site (Table 1) are critical to understanding how organisms interface with geochemical processes. Microbial mat and or solid phase was sampled aseptically, placed on dry ice, and stored at −80°C until DNA extraction.

### Geochemical Analysis

Parallel samples of bulk aqueous phase (<0.2 µm) and sediment intimately associated with the microbial community were obtained simultaneously and analyzed using a combination of field and laboratory methods. As described in more detail in other reports [13], [17], [72], pH, temperature and other redox sensitive species (i.e., Fe^2+^/Fe^3+^; As^III^/As^V^; total dissolved sulfide; dissolved O_2_) were determined using field methods. Total dissolved ions were determined using inductively coupled plasma (ICP) spectrometry and ion chromatography (for all major cations, anions and trace elements). Dissolved gases (CO_2_, H_2_, CH_4_) were determined using closed head-space gas chromatography [13] of sealed serum-bottle samples obtained in the field. A complete dataset of geochemical information corresponding to these sites is available from the YNP Research Coordination Network database (www.rcn.montana.edu). Sediment and microbial mat samples were analyzed using scanning electron microscopy (Phillips Field Emission-SEM, FESEM)) combined with energy dispersive analysis of x-rays (EDAX), back-scatter electron (BSE) detection and electron diffraction (ED).

### DNA Extraction and Library Construction

A standard DNA extraction protocol was used for all samples to avoid variation in composition across sample type due to extraction method. Briefly, 0.5–1 g wet samples were extracted with Proteinase K (final concentration 1 mg/ml) and SDS (final concentration 0.3%) for 0.5 hour at 37°C. After separation of this lysate, the samples were re-extracted using bead-beating protocols. The lysates were combined and extracted with phenol-chloroform and final DNA re-precipitated in ethanol, treated with RNase and gel quantified. DNA was randomly sheared via nebulization, end-polished with consecutive BAL31 nuclease and T4 DNA polymerase treatments, and size-selected using gel electrophoresis on 1% low-melting-point agarose. After ligation to BstXI adapters, DNA fragments were purified, then inserted into BstXI-linearized, medium-copy pBR322 plasmid vectors. The resulting library was electroporated into *E. coli* resulting in high-quality random plasmid libraries with few clones without inserts, and no clones with chimeric inserts [5]. Clones were sequenced from both ends to produce pairs of linked sequences representing ∼820 bp at the end of each insert.

### Random Shotgun Sequencing and Sequence Assembly

Two 384-well cycle-sequencing reaction plates were prepared from each plate of plasmid template DNA for opposite-end, paired-sequence reads according to previously published protocols [5]. A total of 71837 mated sequencing reads (average trimmed sequence read length of 800 bp) were generated from the five sites with an average of 14367 reads per site.

Metagenomic assembly was conducted with the Celera Assembler (Version 4.0 [5], [94]) with the following parameters: doOverlapTrimming = 0, doFragmentCorrection = 0, globalErrorRate = 12, utgErrorRate = 150, utgBubblePopping = 1, and useBogUnitig = 0.

### Sequence Accession Numbers

All individual sequence reads and assembled contigs have been deposited with NCBI under the GenomeProject database (ID #41119) and are assigned a registered locus tag prefix of ‘YNPJCVI’.

### Phylogenetic Analysis of Metagenome Sequence

Phylogenetic classification of individual fragments and contigs was accomplished using several different approaches including (i) phylogenetic binning of individual sequence reads (blastn) to closest reference genomes, (ii) fragment recruitment of individual sequence reads to an extensive library of reference genomes, and (iii) non-similarity based statistical methods (nucleotide word frequency scatter-plots) of sequence assemblies [5]. Metagenome sequence reads (i.e. ∼800 bp) with E-values<10^−10^ compared to a reference genome were then further categorized based on percent nucleotide identity ranging from 47–100%. Only a handful of microbial genomes currently serve as appropriate references for the indigenous organisms within these communities, consequently, the blastn approach provides a quick and useful phylogenetic summary of individual environmental sequence reads. Genome-level phylogenetic analysis was accomplished using fragment recruitment of environmental sequence reads to reference microbial genomes [5]. At the time of writing, the database contained ∼741 completed microbial genomes and 713 draft genomes, including the partial genomes of *Metallosphaera* sp. str. MK1 and *Acidilobus sulfurireducens* str. 18D70. Assembled metagenome sequence data was also analyzed using three dimensional PCA plots of nucleotide word frequencies with a simultaneous phylogenetic classification based on an Automated Phylogenetic Inference System (APIS) described briefly elsewhere [95], or a blast based classification [5]. Briefly, APIS is a system for automatic creation and summarizing of phylogenetic trees for each protein encoded by a genome or metagenomic dataset. Metagenome sequence reported here can be viewed with these utilities at http://gos.jcvi.org/openAccess/scatterPlotViewer.html.

### Functional Analysis of Metagenome Sequence

We built a custom reference sequence database that covers all pathways and enzymes represented in the MetaCyc [45] pathway database by combining the protein sequences that are distributed with MetaCyc (including sequences for which only cross references to external databases were provided) with EC-number associated sequences from the SwissProt Enzyme database [46]. The resulting 168,000 sequences were clustered at 95% identity using CD-HIT [96] to yield a database of over 94,000 protein sequences. Each of the sequences was associated with individual reactions in MetaCyc either through the direct associations in MetaCyc or though full/partial EC numbers.

Partial gene sequences were predicted from unassembled shotgun sequencing reads using an approach that combined evidence from multiple sources using the Evigan consensus gene prediction method [49]. All candidate ORFs on a metagenomic sequence read were first predicted based on stop codons found on all six frames and allowing for run-on in order to include partial ORFs. Candidate ORF translations were then annotated using blastp searches against the NCBI non-redundant protein database and FastHMM (http://www.microbesonline.org/fasthmm/) searches against Pfam [97] and Superfamily [98] domain databases. De novo ORF predictions were also made using three prokaryotic gene finding tools: Glimmer [99], Prodigal (http://compbio.ornl.gov/prodigal/), and Metagene [100]. The evidence from the blast/FastHMM searches and de novo gene finders was then combined in an unsupervised manner using Evigan. The consensus gene prediction was performed by first binning reads based on GC content and then running Evigan on each 10,000 read bin separately.

The predicted protein translations were clustered using CD-HIT at 97% identity using default parameters, and representative sequences from each cluster were compared against the custom MetaCyc associated protein database described above using blastp. For each metagenomic query translation all hits to reference proteins with blast bit scores above 50 and within 20% of the top hit bit score were retained for further analysis. Reference sequences that were only associated with reactions though partial EC numbers were only included in the filtered hit list for each query sequence unless no other sequences had blast bit scores above 50. Blastp hit counts for each reaction and metagenome were obtained by counting the number of unique metagenomic translations whose filtered hit list included sequences associated with the reaction. Finally, pathway activity scores for each MetaCyc pathway were obtained by calculating the median hit count for reactions in a given pathway.

To facilitate comparison of reference genomes and metagenomes, we also derived a pathway completeness score indicating the percentage of biochemical steps in a pathway found in each metagenome. Since many reactions occur in multiple pathways and thus are not reliable indicators of the presence of a specific pathway, the completeness score was calculated to account for the pathway specificity of a given reaction. Additionally, MetaCyc includes many alternate pathway variants that only differ by one or two reactions, and these minor variants should not be counted as completely separate pathways when pathway specificity of a reaction is calculated. The pathway specificity weight for each reaction i was calculated as
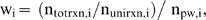
where n_pw,i_ is the number of distinct pathways that have reaction i, n_totrxn,i_ is the total number of reactions in all pathways that have this reaction, and n_unirxn,i_ is the number of unique reactions in all pathways that have this reaction. Note that for identical pathways, n_totrxn,i_/n_unirxn,i_ = n_pw,i_, i.e. there is no penalty if the reaction appears in multiple identical pathways. The pathway completeness score for each pathway j was then calculated as
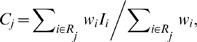
where *R_i_* is the set of reactions in pathway *j*, and *I_i_* = 1 if reaction i has at least one hit in a given metagenome or genome and zero otherwise.

PCA analysis of pathway completeness and activity scores was performed using Matlab (Mathworks, Inc.) after filtering out pathways with less than 5 reactions for completeness-score based analysis. Pathways for Figure 4 were selected by rank ordering all reactions based on their contribution to the top three principal components and picking the top 15 reactions that most contribute to variability across genomes/metagenomes. Redundant pathways that differ by only one reaction were removed from the clustering data set manually. Hierarchical average linkage clustering of data for selected pathways was performed using MeV v4.2 [101] with Euclidean and Pearson correlation as distance metrics for completeness and activity scores, respectively.

The assembled environmental sequence data was also screened for specific functional genes corresponding to known and or putative pathways in material and energy transfer. Query DNA sequences known to code for proteins important in the oxidation of reduced chemical constituents or the reduction of a terminal acceptor (Table S3) were used to search (WU-tblastn) the metagenome sequence. Environmental sequence fragments exhibiting homology (E-values<10^−10^) to query sequences were then reanalyzed using NCBI-blastp against the nr database, and carefully assessed individually using phylogenetic analysis of deduced protein sequences against known relatives, as well as fragment length relative to query length (Tables S3–S4). False positives were eliminated by this screening process and included (i) sequences matching the correct protein family of the query sequence, but not the exact query sequence (e.g., Mo-pterin oxidoreductases versus a specific protein within this family), (ii) sequences that match a query sequence due to homologous regions, but are clearly associated with a gene or gene cluster with different function, and (iii) sequences that returned mis-annotated NCBI-blastp relatives. It is also possible that our inventory of metabolic potential has missed sequences related to a specific query gene. For example, some genes found in the metagenome data were of insufficient length relative to a specific query sequence to make a definitive assignment. Moreover, the lower coverage depth for some sites (especially NGB) suggests that any functional analyses should be considered a preliminary assessment of metabolic potential.

## Supporting Information

Table S1 Summary of assembly statistics obtained for each of the five chemotrophic geothermal springs located in Yellowstone National Park.
(0.05 MB DOC)

Table S2 Summary of 16S rRNA gene sequences observed in assembled metagenome sequence data from five chemotrophic environments.
(0.06 MB DOC)

Table S3 List of gene sequences and corresponding accession numbers used to query the assembled environmental sequence data for assessing potential metabolic attributes associated with the predominant phylotypes found in each of the five geothermal sites. Subsequent environmental sequence hits with E-values less than 10-5 were analyzed in detail to identify ‘high-confidence’ putative gene sequences important in C fixation and electron transfer.
(0.06 MB XLS)

Table S4 Metagenome sequence hits from five geothermal sites (Crater Hills = yellow; Norris Geyser Basin = red; Joseph's Coat Hot Springs = blue; Mammoth Hot Springs = green; Calcite Springs = violet) exhibiting significant similarity to ‘query’ gene sequences known to code for proteins involved in C fixation, electron transfer, and detoxification (shown in gray and referenced separately in Table S3).
(0.21 MB XLS)

Figure S1 Phylogenetic tree of archaeal 16S rRNA gene sequences from Crater Hills (CH-AS, yellow), Norris Geyser Basin, (NGB-BE, red) and Joseph's Coat Hot Springs (JCHS, blue) including (i) clones obtained using standard PCR protocols and universal archaeal primers, and (ii) assembled environmental sequence data (labeled with Meta; also see Supplemental Table S3). [The percent of sequenced clones obtained using PCR relative to the total for each site is given in parentheses. The fragment length for sequences obtained from metagenome data is given in parentheses. Isolates in black bold type; **  =  full genome sequence; *  =  partial genome sequence; neighbor joining tree, boot strap values are per 1000]).
(0.76 MB TIF)

Figure S2 Fragment recruitment of YNP metagenome sequence to the genome of Pyrobaculum spherical virus (PSV). Assembly of viral sequence reads from Joseph's Coat Hot Springs (blue) ranging from ∼70–80% identity to PSV resulted in ∼2–3x coverage relative to the reference viral genome.
(0.06 MB TIF)

Figure S3 Functional grouping of metagenomes and genomes using PCA analysis of MetaCyc pathway recruitment data. A. PCA analysis of pathway completeness scores for five metagenomes and five reference genomes (see caption for Figure 4 for reference genome designations). Projection into the first two principal components is shown. Key pathways that contribute to the two components are shown in a biplot format. B. PCA analysis of pathway activity scores for metagenomes.
(0.37 MB TIF)
