# Supplementary material for: Metagenomes from High-Temperature Chemotrophic Systems Reveal Geochemical Controls on Microbial Community Structure and Function
Source: PLoS One. 2010 Mar 19;5(3):e9773. doi: 10.1371/journal.pone.0009773 (PMC2841643; doi:10.1371/journal.pone.0009773)
Supplement: Table S1 — Summary of assembly statistics obtained for each of the five chemotrophic geothermal springs located in Yellowstone National Park. (0.05 MB DOC) [file pone.0009773.s001.doc]

| **Parameter** | **Geothermal Site** | | | | |
| --- | --- | --- | --- | --- | --- |
|  | ***Crater Hills*** | ***Norris Geyser Basin*** | ***Joseph’s Coat Hot Springs*** | ***Mammoth Hot Springs*** | ***Calcite Hot Springs*** |
| **Reads Used in Assembly** | 15,149 | 13,356 | 13,943 | 14,371 | 15,018 |
| **Average Read Length (bp)** | 764 | 833 | 810 | 829 | 768 |
| **Total Read Length (bp)** | 11,578,001 | 11,125,453 | 11,296,109 | 11,909,915 | 11,532,459 |
| **Percent of Unassembled Reads** | 6.9 | 39.2 | 23.5 | 4.9 | 20.7 |
| **Percent of Small Contig Reads** | 21.0 | 55.5 | 60.8 | 28.4 | 69.9 |
| **Percent of Large Contig Reads** | 62.1 | 0.7 | 10.8 | 66.1 | 2.9 |
| **Total Small Contigs (< 10 kb)** | 476 | 1912 | 1629 | 190 | 1597 |
| **Base Pairs in Small Contigs** | 871, 167 | 3,575,484 | 3,466,528 | 655,851 | 3,637,753 |
| **Percent of Small Contig Bases** | 45.1 | 99.0 | 89.4 | 38.1 | 97.0 |
| **Total Large Contigs** | 38 | 3 | 30 | 45 | 9 |
| **Base Pairs in Large Contigs >10 kb** | 1,060,746 | 35,931 | 410,443 | 1,066,654 | 112,866 |
| **Percent of Large Contig Bases** | 54.9 | 0.99 | 10.6 | 61.9 | 3.0 |
| **Length of Longest Contig (bp)** | 78,118 | 12,678 | 22,186 | 108, 512 | 19,179 |
| **Mean Contig Length (bp)** |  | 1,886 | 2,337 | 7,330 | 2,335 |
| **Total Scaffolds** | 420 | 1287 | 928 | 99 | 645 |
| **Contigs in Scaffolds** | 514 | 1915 | 1659 | 235 | 1606 |
| **Bases in Scaffolds** | 1,931,913 | 3,611,415 | 3,876,971 | 1,722,505 | 3,750,619 |
| **Length of Largest Scaffold (bp)** | 386,883 | 185,269 | 174,823 | 196,601 | 1,005,040 |
| **Mean Contigs per Scaffold** | 1.2 | 1.5 | 1.8 | 2.4 | 2.5 |
| **Mean Bases per Scaffold** | 4,600 | 2,806 | 4,178 | 17,399 | 5,815 |
| **Contig Coverage** | 5.02 | 1.78 | 2.13 | 6.56 | 2.26 |
| **Read Coverage** | 5.99 | 3.08 | 2.91 | 6.91 | 3.07 |

**Table S1.** Summary of assembly^1^ statistics obtained for each of the five chemotrophic geothermal springs located in Yellowstone National Park.

^1^ environmental sequence reads assembled using the Celera Assembler [5].
