# Supplementary material for: Metagenomes from High-Temperature Chemotrophic Systems Reveal Geochemical Controls on Microbial Community Structure and Function
Source: PLoS One. 2010 Mar 19;5(3):e9773. doi: 10.1371/journal.pone.0009773 (PMC2841643; doi:10.1371/journal.pone.0009773)
Supplement: Table S2 — Summary of 16S rRNA gene sequences observed in assembled metagenome sequence data from five chemotrophic environments. (0.06 MB DOC) [file pone.0009773.s002.doc]

**Table S2.** Summary of 16S rRNA gene sequences^1^ observed in assembled metagenome sequence data from five chemotrophic environments.

| **Geothermal Site** | **NCBI Closest Cultivated Relative^2^** | **Length (bp)** | **Identity** | **NCBI No.** |
| --- | --- | --- | --- | --- |
| **Crater Hills** | *Sulfolobus solfataricus* | 1552 | 91% | X03235 |
| *pH= 2.5, T= 74-76* | *Caldococcus noboribetus* | 494 | 96% | D85038 |
|  | *Acidilobus saccharovorans* | 437 | 97% | AY350586 |
|  | *Acidianus infernus* | 248 | 98% | X89852 |
| **Norris Geyser Basin** | *Nitrosocaldus yellowstonii* strain HL72 | 1507 | 84% | EU239960 |
| *pH= 3.0, T= 65-68* | *Staphylothermus marinus* | 1327 | 85% | X99560 |
|  | *Anaerobaculum mobile* | 676 | 85% | AJ243189 |
|  | *Staphylothermus achaiicus* (Isolate P8) | 544 | 84% | AJ012645 |
|  | *Geogemma indica* strain 296 1 | 355 | 82% | DQ492260 |
|  | *Hydrogenobaculum* sp. Y04AAP1 | 241 | 98% | CP001130 |
| **Joseph's Coat Hot Springs** | *Pyrobaculum calidifontis* JCM 11548 | 1286 | 95% | AB078332 |
| *pH= 6.1, T= 80-82* | *Sulfolobus solfataricus* | 778 | 91% | X03235 |
|  | *Pyrobaculum islandicum* DSM 4184 | 498 | 93% | CP000504 |
|  | *Vulcanisaeta distributa* | 415 | 99% | AB063639 |
|  | *Thermoproteus neutrophilus* V24Sta | 351 | 98% | CP001014 |
|  | *Caldivirga maquilingensis* IC-167 | 229 | 97% | AB013926 |
| **Mammoth Hot Springs** | *Sulfurihydrogenibium* sp. Y03AOP1 | 1518 | 98% | CP001080 |
| *pH= 6.6, T= 70-72* |  |  |  |  |
| **Calcite Springs** | *Sulfurihydrogenibium* sp. Y03AOP1 | 1518 | 98% | CP001080 |
| *pH= 7.8, T= 74-76* | *Thermocrinus ruber* | 689 | 98% | AJ005640 |
|  | *Thermus aquaticus* YT-1 | 608 | 98% | NR_025900 |
|  | *Geothermobacterium ferrireducens* str. FW-1a | 581 | 99% | AF411013 |
|  | *Sulfurihydrogenibium* sp. Y03AOP1 | 533 | 99% | CP001080 |
|  | *Thermotoga lettingae* TMO | 176 | 94% | CP000812 |
|  |  |  |  |  |

^1^ only rRNA gene sequences with fragment lengths greater than 200 bp are reported here with exception of the *Thermotoga*-like 16S sequence at Calcite Springs; ^2^ closest cultivated relatives are provided here, but these specific genus species must not be over-interpreted as strict phylogenetic assignments (especially when fragment lengths are low relative to the full-length 16S rRNA gene sequence of ~ 1500 bp).
