# Supplementary figures and images for: Metagenomes from High-Temperature Chemotrophic Systems Reveal Geochemical Controls on Microbial Community Structure and Function
Source: PLoS One. 2010 Mar 19;5(3):e9773. doi: 10.1371/journal.pone.0009773 (PMC2841643; doi:10.1371/journal.pone.0009773)

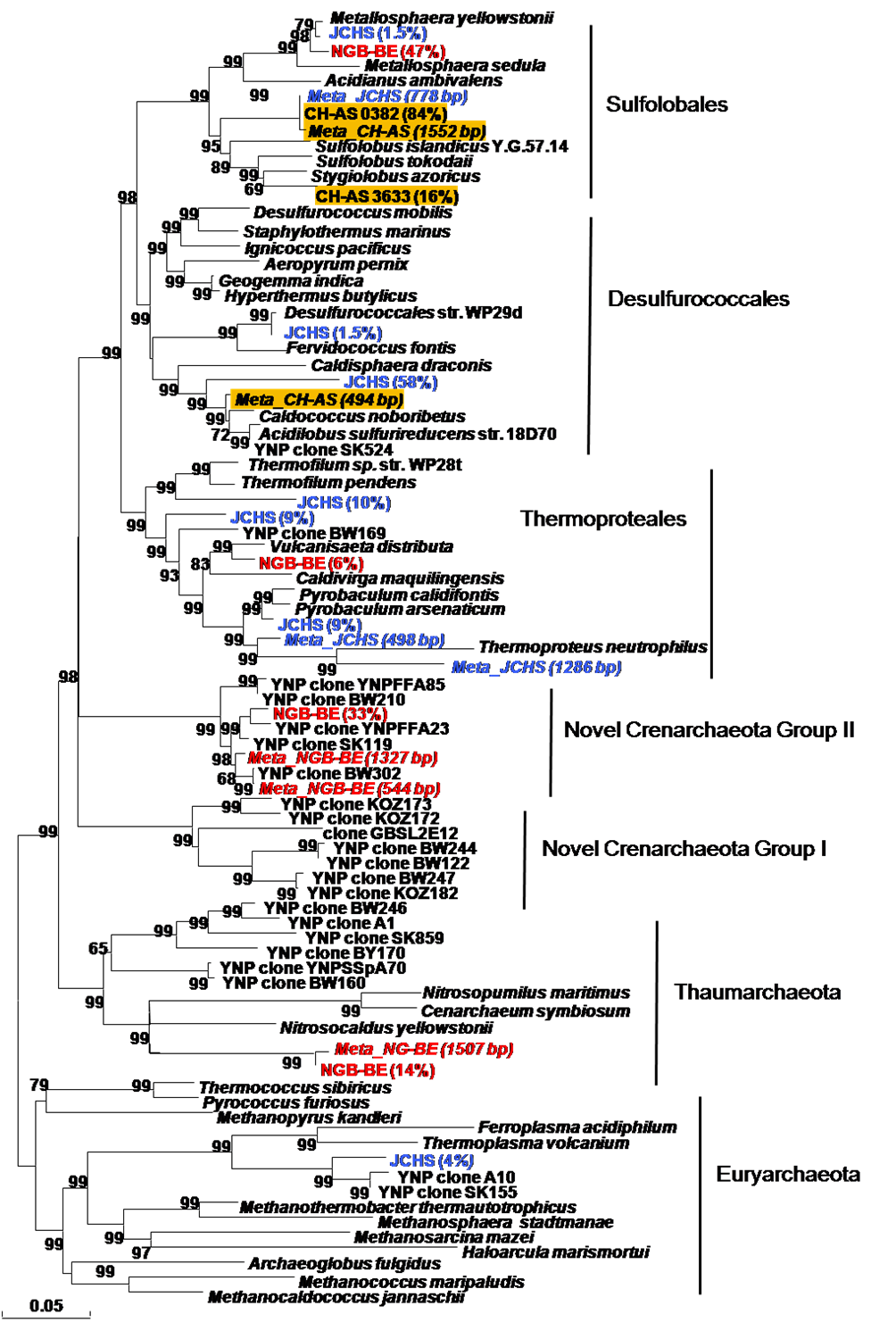

Supplement: Figure S1 — Phylogenetic tree of archaeal 16S rRNA gene sequences from Crater Hills (CH-AS, yellow), Norris Geyser Basin, (NGB-BE, red) and Joseph's Coat Hot Springs (JCHS, blue) including (i) clones obtained using standard PCR protocols and universal archaeal primers, and (ii) assembled environmental sequence data (labeled with Meta; also see Supplemental Table S3). [The percent of sequenced clones obtained using PCR relative to the total for each site is given in parentheses. The fragment length for sequences obtained from metagenome data is given in parentheses. Isolates in black bold type; ** = full genome sequence; * = partial genome sequence; neighbor joining tree, boot strap values are per 1000]). (0.76 MB TIF) [file pone.0009773.s005.tif]

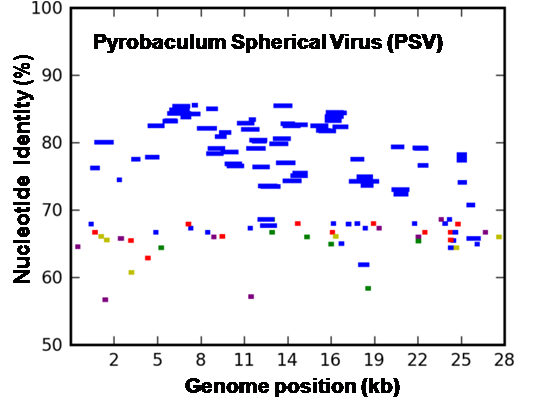

Supplement: Figure S2 — Fragment recruitment of YNP metagenome sequence to the genome of Pyrobaculum spherical virus (PSV). Assembly of viral sequence reads from Joseph's Coat Hot Springs (blue) ranging from ∼70–80% identity to PSV resulted in ∼2–3x coverage relative to the reference viral genome. (0.06 MB TIF) [file pone.0009773.s006.tif]

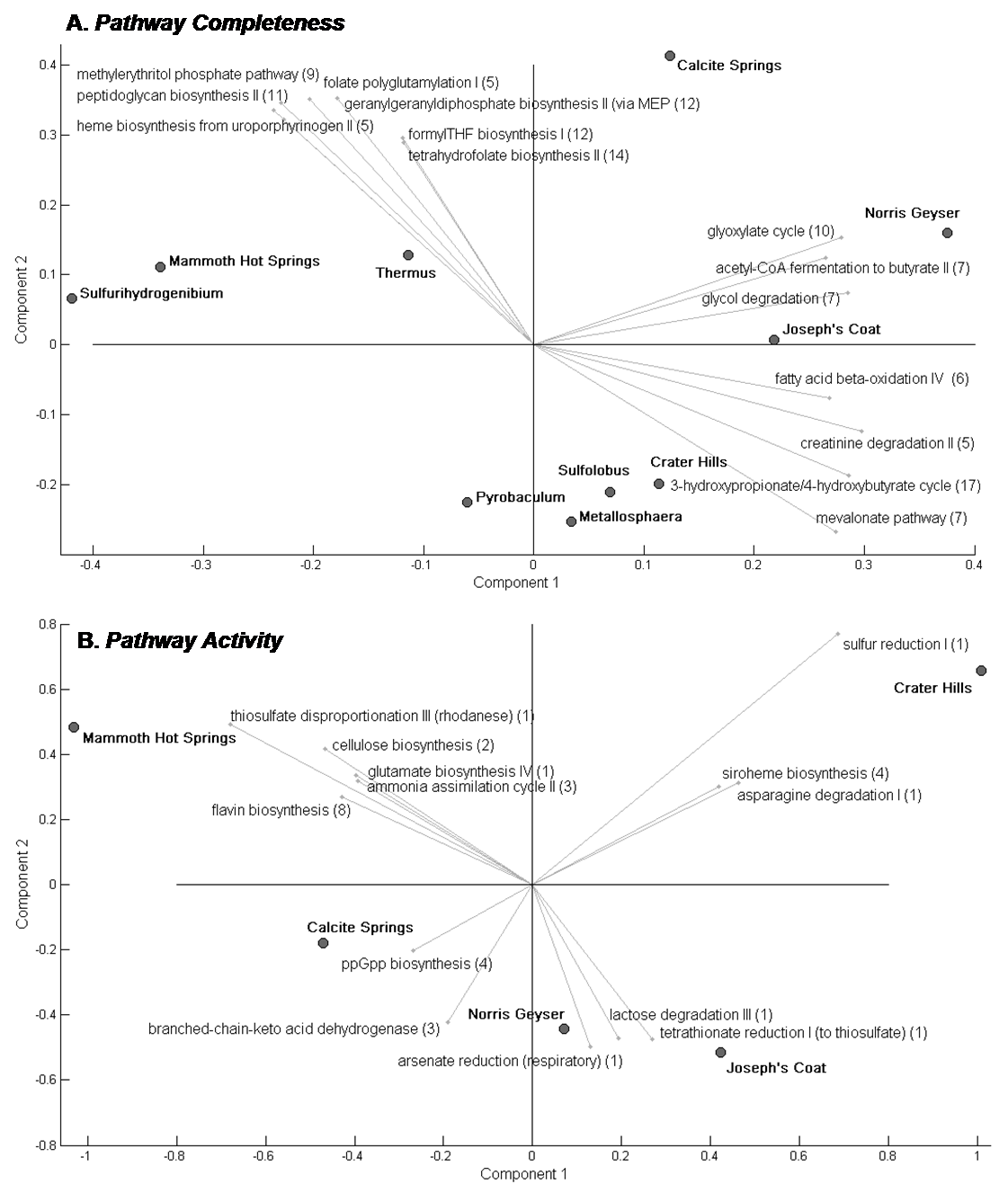

Supplement: Figure S3 — Functional grouping of metagenomes and genomes using PCA analysis of MetaCyc pathway recruitment data. A. PCA analysis of pathway completeness scores for five metagenomes and five reference genomes (see caption for Figure 4 for reference genome designations). Projection into the first two principal components is shown. Key pathways that contribute to the two components are shown in a biplot format. B. PCA analysis of pathway activity scores for metagenomes. (0.37 MB TIF) [file pone.0009773.s007.tif]
